# Supplementary material for: Screening of In Vitro Heavy Metal Tolerance in Tocoyena brasiliensis Mart. (Rubiaceae)
Source: Plants (Basel). 2025 Apr 28;14(9):1331. doi: 10.3390/plants14091331 (PMC12073447; doi:10.3390/plants14091331)
Supplement: Supplementary file 1 [file plants-14-01331-s001.zip › plants-3496037-supplementary.pdf]

Supplementary Materials

Table S1. One-way ANOVA used to test the effect of metal concentration (mg L^{-1} of Zn, Pb and Cd separately) for the studied biometric traits. The *P*-values in bold indicate significant differences.

	Zn	Pb	Cd
Survival	**<0.0001**	0.43068	0.06234
Shoot number	0.32212	0.06234	0.32212
Shoot length	**<0.0001**	**<0.05**	0.81379
Leaf number per shoot	**<0.0001**	**<0.001**	**<0.01**
Callus formation	**<0.0001**	**<0.0001**	0.10106

Zn: 0, 50, 100, 200, and 400 mg L^{-1} zinc acetate; Pb: 0, 50, 100, 200, and 400 mg L^{-1} lead acetate; Cd: 0, 2, 4, 6, and 8 mg L^{-1} cadmium chloride.

Table S2. Correlation coefficients between heavy metal (Zn, Pb and Cd separately) and biometric traits.

	Zn	Pb	Cd
Survival	-0.88	-0.36	-0.43
Shoot number	-0.33	-0.10	0.45
Shoot length	-0.88	-0.55	-0.26
Leaf number per shoot	-0.91	-0.64	-0.67
Callus formation	-0.71	-0.96	-0.26
